# Welcome to *Environmental Microbiome*

**DOI:** 10.1186/s40793-019-0340-8

**Published:** 2019-04-01

**Authors:** Joy E. M. Watts

**Affiliations:** University of Portsmouth, University House, Winston Churchill Ave, Portsmouth, PO1 2UP UK

I am delighted to serve as Editor-in-Chief for *Environmental Microbiome*, sister publication to *Microbiome* and *Animal Microbiome*. We are kicking off *Environmental Microbiome* with a research article by Barbara Cania and colleagues: A long-term field experiment demonstrates the influence of tillage on the bacterial potential to produce soil structure-stabilizing agents such as exopolysaccharides and lipopolysaccharides [1]. This article looks at how the common agricultural practice of tilling can disrupt the potential of bacteria to produce the natural compounds that ‘glue’ soil particles together – this ‘gluing’ effect helps prevent soil erosion.

Environmental microbiology can be loosely defined as the study of microbes, their functions, and interactions in all habitats on Earth (and beyond). This is a burgeoning research area that covers air, soil and aquatic systems, astrobiology, biogeochemical cycles, plant-soil interactions, extreme environments, and spatial, temporal and perturbation studies. Unfortunately, due to the non-culturability of most environmental species, our understanding of microbial community composition and function is somewhat driven by technological innovation. A proliferation of 16S rRNA gene studies in the 1990s started to reveal the diversity present in environmental systems. The increasing ease and availability of sequencing-based approaches enabled larger fragments of environmental DNA to be cloned [2]. This was followed shortly after by early metagenomics studies such as Rondon et al. 2000 [3], and genome reconstruction from environmental samples [4]. These omics-based tools have enabled new insights into microbial diversity, function, and the complex ecological interactions therein. This is an exciting time and continuing innovations in sequencing technology and downstream analysis have now made assessments of complex environmental microbiomes commonplace and also revealed how much diversity is left to discover!

This journal will provide a forum to publish hypothesis-driven environmental microbiome studies such as marker gene surveys, metagenomes, metatranscriptomes, metaproteomes, metabolomes, microbial genome reconstruction, and single-cell analysis from environmental habitats. Previously, many studies have focused on bacterial and archaeal microbiome analysis, but to obtain a more holistic view of ecological interactions we should aim to include the environmental mycobiome and virome to better understand different systems. Understanding the function and activities of microbial dark matter and the rare biosphere will be a major focus for the journal, and utilising improved technologies such as single-cell sequencing analysis will also provide valuable insights. New methodologies, theoretical community analysis, and development of bioinformatics tools are also welcome due to their importance in moving our understanding forward.

The journal is transitioning from *Standards in Genomic Sciences* and, as such, we are in the fortunate position of already having an Impact Factor that we can build upon. The Editorial team for this journal is diverse and reflects the broad research areas that will be reflected in this publication. As a sister journal to *Microbiome* and *Animal Microbiome* the same high standards of openness and transparency will be employed. To promote open access science, which is crucial for ensuring the most impact from published research, all articles published in *Environmental Microbiome* will be freely available through the journal web portal and within major research publication databases such as PubMed Central. Also, because authors, not the publisher will hold the copyright to their work, they will be free to distribute published articles.

The editorial team at *Environmental Microbiome* hope that you will support this endeavour by submitting your research studies and furthering our understanding of environmental microbiology.

## Authors’ information

Joy Watts is the Editor-in-Chief of *Environmental Microbiome* and a Senior Editor of *Microbiome*.
